# Knowledge, attitude, and perception of Pakistani women regarding breast cancer and mammography screening: A cross-sectional study

**DOI:** 10.1097/MD.0000000000042806

**Published:** 2025-06-06

**Authors:** Qasim Mehmood, Abdul Rafay, Hafiza Qurat Ul Ain, Muhammad Junaid Tahir, Abdullah Saeed, Abdul Hanan, Zohaib Yousaf, Zair Hassan, Khabab Abbasher Hussien Mohamed Ahmed, Muhammad Sohaib Asghar

**Affiliations:** a King Edward Medical University, Lahore, Pakistan; b Queen Alexandra Hospital Portsmouth University NHS Trust, Cosham, Portsmouth, UK; c CMH Multan Institute of Medical Sciences, Multan, Pakistan; d Pakistan Kidney and Liver Institute and Research Center (PKLI and RC), Lahore, Pakistan; e Lahore General Hospital, Lahore, Pakistan; f Tower Health, Reading, PA; g Lady Reading Hospital, Peshawar, Pakistan; h Faculty of Medicine, University of Khartoum, Khartoum, Sudan; i Mayo Clinic, Rochester, NY.

**Keywords:** breast, carcinoma, imaging, mammography, mortality, public health

## Abstract

Breast cancer is one of the most common cancers in females. In low- and middle-income countries like Pakistan, reported incidence and prevalence remain unreliable. The aim was to evaluate Pakistani women’s knowledge, attitude, and perception of breast cancer. This cross-sectional study was conducted among Pakistani women from Punjab, Pakistan. A self-administered online questionnaire was formulated to collect data from the participants from September 24, 2022, to October 24, 2022. The questionnaire included sociodemographic data, knowledge questions regarding breast cancer risk factors, knowledge questions about symptoms of breast cancer, and questions assessing the participants’ attitudes toward mammography screening. For knowledge questions, a score of more than 7 out of 13 was considered good knowledge. For attitude questions, regression analysis was done and a score of more than 5 out of 11 was considered a positive attitude. Data was analyzed using SPSS version 25 (Chicago). 796 women participated in this study. Out of 796 participants, 543 (68.2%) were between 20 and 30 years of age, and 43 (5.4%) were more than 40 years old. The level of knowledge regarding breast cancer risk factors was suboptimal. 523 (65.7%) of the participants thought that breast cancer risk increases with advancing age and 218 (27.4%) thought that early menarche increases the risk of developing breast cancer. 471 (59.2%) mentioned that mammography is an effective tool for detecting breast cancer, and 30 (3.8%) thought that there is a cure for breast cancer. According to regression analysis, higher education, higher monthly household income, and married marital status were significantly associated with good knowledge related to breast cancer. Although most of the population is aware of breast cancer’s risk factors and symptoms, they avoid screening such as mammography. Public awareness projects could help increase women’s knowledge of breast cancer signs, symptoms, and screening.

## 1. Introduction

Breast cancer has the highest global incidence in women, overtaking lung cancer. The number of individuals diagnosed with cancer worldwide has doubled, from around 10 million in 2000 to 19.3 million in 2020. In 2020, breast cancer accounted for 2.26 million cancer cases.^[1]^ In low- and middle-income countries (LMICs) like Pakistan, figures remain unreliable due to poor reporting and scarcity of diagnostic and treatment facilities.^[1]^

The incidence of breast cancer is increasing globally to more than 2 million cases reported annually. This burden is much greater in LMICs like Pakistan due to limited access to screening and diagnostic services, as well as access to healthcare and treatment.^[2]^ According to a study done by Tufail et al,^[3]^ the percentage of breast cancer cases in Pakistan was 24.1% among a sample of more than 40 thousand cancer patients. In another study done by Tufail et al,^[4]^ among a total of 1,11,941 cancer patients breast cancer was the most prevalent one (22.2%). According to the global cancer observatory published by WHO,^[5]^ the incidence and 5-year prevalence of breast cancer cases in Pakistan is 16.5% and 18.9% respectively.

Breast cancer is a multifactorial disease. Around 95% of cancers are associated with genetic mutations that result from environmental or lifestyle factors. The remaining percentage is related to inherited genes like Breast Cancer gene 1 and Breast Cancer gene 2 (BRCA1/BRCA2).^[6]^ Increasing age, positive family history, reproductive factors such as early menarche, late menopause, late age at first pregnancy, and low parity, both endogenous and exogenous estrogen in the form of oral contraceptives and hormone replacement therapy and sedentary lifestyle are important risk factors of breast cancer.^[7]^ Screening plays a significant role in the prevention and early detection of breast cancer, with up to a 20% reduction in mortality. Early-stage breast cancer diagnosis carries a > 90% 5-year survival rate, compared to around 15% of advanced-stage cancer.^[8]^ Survival rates are much higher in European countries than in LMICs, attributed to significantly better screening and treatment facilities. breast self-examination, clinical breast examination, and mammography are well-recognized screening methods for breast cancer.^[4]^

Despite the benefits of mammography, several previous studies have shown poor knowledge, attitudes, and utilization of screening services among various research populations in LMICs. A study conducted was by Bhargava et al^[9]^ to identify possible barriers to mammographic screening among Pakistani women in Norway. They have concluded that various factors play a role in shaping their preference to attend breast cancer screening program. Some of the factors were their lack of trust in the healthcare system, gender of the radiographer, religious beliefs, financial problems, lack of access to screening centers, and personal preferences. There is no national breast cancer screening program in Pakistan. The use of mammography for breast cancer screening is limited.^[1]^

This study aims to evaluate breast cancer knowledge among Pakistani women, including risk factors, signs and symptoms, and early detection methods, especially mammography screening. It also aims to explore the associations between women’s knowledge about breast cancer and their breast cancer screening practice. The findings of this study will identify the critical barriers to mammographic screening of breast among women in order to devise a plan to promote breast cancer screening awareness among them.

## 2. Materials and methods

### 2.1. Study design and sampling procedures

This was a cross-sectional study analyzing data from the female population of Punjab, Pakistan, to assess their knowledge, attitude, and perception regarding breast cancer and mammography screening. Sampling was done by convenience sampling technique.

### 2.2. Study participants

Data was collected from 796 participants from Punjab, Pakistan. The study included only female participants aged more than 15 years who were in the contact list of the investigators or were coming to outpatient departments (OPDs) or clinics where the investigators were working. No specific criteria were implied for the respondents to participate in the study.

### 2.3. Data collection procedure

A self-administered online questionnaire was formulated to collect data from the participants. The questionnaire included sociodemographic data, knowledge questions regarding breast cancer risk factors, knowledge questions about symptoms of breast cancer, and questions assessing the participants’ attitudes toward mammography screening. The questionnaire employed the 3-point Likert scale. Data collection was started by sharing questionnaires via Facebook, WhatsApp, Messenger, etc. Only 1-time submission was allowed by the investigators to avoid duplicate responses. Data collection started on September 24, 2022 and was open to participants for 1 month till October 24, 2022. Female patients coming to outpatient departments (OPDs), wards, and clinics where the investigators worked were also included in the study.

### 2.4. Ethical considerations and consents

The formal ethical approval for the study was obtained from Lady Reading Hospital, Peshawar (Ref. No. 230/LRH/MTI). The study protocol and format of consent were reviewed by the ethical committee of the Lady Reading Hospital which approved that informed consent was needed from participants only, considering the study only involved minimal risk. In other words, consent from parents/guardians was waived. The questionnaire was kept anonymous, and no questions were asked of the participants revealing their identity. Informed consent was implied for all the study participants, whether responding to online Google forms or giving data to investigators during their visits to their OPDs and clinics. Complete confidentiality and privacy of the collected data were ensured. The study followed the ethical standards suggested by the Helsinki Declaration (Revised 2013) and the International Ethical Guidelines for Human Research in Health (2016). The study also followed the STROBES (Strengthening the Reporting of Observational Studies in Epidemiology) statement guidelines for conducting cross-sectional studies.

### 2.5. Data analysis

The data from Google Forms was used to generate a Microsoft Excel sheet and was then analyzed by the software Statistical Package for the Social Sciences (SPSS) version 25 (Chicago). Quantitative variables were reported as frequency and percentages. A chi-square test was applied to compare the qualitative variables. Univariate and multivariate logistic regression analyses were performed to determine the factors associated with good knowledge about breast cancer and its screening among the study participants. The total score of the knowledge questions was 13, and a score of more than 7 was considered good knowledge. For attitude questions, regression analysis was done to determine factors associated with a positive attitude about mammography screening among the participants. There were 11 attitude questions, and each correct response carried 1 mark. A score of more than 5 was considered a positive attitude.

### 2.6. Questionnaire

A thorough literature review was conducted before the questionnaire was implemented.^[10–12]^ We also adhered to the CHERRIES guidelines to improve the reporting of our online questionnaire.^[13]^ After receiving feedback from doctors with relevant research experience, the questionnaire was subsequently finalized. The reliability coefficient, a gauge of the questionnaire’s internal consistency, was evaluated using Cronbach Alpha. The questionnaire’s Cronbach alpha coefficient was 0.83, as reported by Alam et al.^[14]^ An overview of the study’s goals and objectives was provided in the first paragraph of the questionnaire, which was then divided into 4 sections that assessed participants’ knowledge, attitudes, and practices related to breast cancer.

## 3. Results

### 3.1. Basic characteristics and demographics of the participants

Out of 796 participants, 543 (68.2%) were between 20 and 30 years of age, and 43 (5.4%) were more than 40 years old. 26 (3.3%) had a level of education from primary up to high school level, and 145 (8.2%) were postgraduates. 80 (10.1%) were urban residents while 716 (89.9%) were from a rural setting. 182 (22.9%) were working women, 135 (17.0%) were housewives and 479 (60.2%) were students. 573 (71.9%) of the study participants were single, 196 (24.6%) were married and 28 (3.5%) were separated/widows. 37 (4.6%) of the participants got married under the age of 20, 172 (21.6%) got married between the ages of 20 and 30 years, and 15 (1.9%) got married after 30 years of age. 151 (19.0%) participants had children and 645 (81.0%) didn’t have children.

The age of menarche among 140 (17.6%) participants was <12 years, 542 (16.8%) had their age of menarche between 12 and 14 years of age, and it was above 14 years in 114 (14.3%) participants. 24 (3%) were menopausal and 772 (97%) were non-menopausal. 106 (13.3%) participants had a monthly household income of <25,000 PKR (approximately 100 US Dollars), and 276 (34.7%) had income above 100,000 PKR (approximately 400 US Dollars). Family history of breast cancer was positive in 164 (20.6%) participants while 726 (79.4%) did not have a positive family history (Table 1).

**Table 1:** General characteristics and demographics of the study participants (n = 796).

Variables	Characteristics	Descriptive/frequency
Age (yr, mean = 25.92 ± 6.81)	<20	99 (12.4%)
	20–30	543 (68.2%)
	30–40	111 (13.9%)
	>40	43 (5.4%)
Education	Primary up to matric	26 (3.3%)
	Up to secondary (intermediate)	115 (14.4%)
	Bachelors	510 (5.4%)
	Postgraduate	145 (18.2%)
Residence	Urban	80 (10.1%)
	Rural	716 (89.9%)
Occupation	Working women	182 (22.9%)
	Housewife	135 (17.0%)
	Student	479 (60.2%)
Marital status	Single	572 (71.9%)
	Married	196 (24.6%)
	Widow/separated	28 (3.5%)
Age of marriage	<20 yr	37 (4.6%)
	20–30 yr	172 (21.6%)
	>30 yr	15 (1.9%)
Having children?	Yes	151 (19.0%)
	No	645 (81.0%)
Age of menarche	<12 yr	140 (17.6%)
	12–14 yr	542 (68.1%)
	>14 yr	114 (14.3%)
Menopause?	Yes	24 (3.0%%)
	No	772 (97.0%)
Monthly household income (PKR)	<25,000	106 (13.3%)
	25,000–50,000	129 (16.2%)
	50,000–75,000	137 (17.2%)
	75,000–100,000	148 (18.6%)
	>100,000	276 (34.7%)
Any previous breast symptoms like (discharge, pain, itching, etc)	Yes	127 (16.0%)
	No	669 (84.0%)
Any current breast symptoms like (discharge, pain, itching, etc)	Yes	70 (8.8%)
	No	726 (91.2%)
Family history of breast cancer?	Yes	164 (20.6%)
	No	726 (79.4%)

PKR = Pakistani rupee.

### 3.2. Knowledge about risk factors of breast cancer

Five hundred twenty three (65.7%) of the participants thought that breast cancer risk increases with advancing age, 97 (12.2%) did not think that, and 176 (22.1%) were not sure. 329 (41.3%) believed that obesity increases the risk of developing breast cancer, 268 (33.7%) did not think that, and 199 (24.0%) were not sure. 300 (37.7%) thought that irritation of tight brassiere could increase breast cancer risk, and 188 (23.6%) thought that having larger breasts increases the risk of developing breast cancer. 524 (65.8%) thought that alcohol consumption increases the risk of developing breast cancer, and 581 (73.0%) thought that cigarette smoking increases the risk of developing breast cancer.

Two hundred eighteen (27.4%) thought that early menarche increases the risk of developing breast cancer, 254 (31.9%) thought that late menopause increases the risk of developing breast cancer, 559 (70.2%) thought that not breastfeeding increases the risk of developing breast cancer, and 641 (80.5%) thought that positive family history increases the risk of developing breast cancer. 400 (50.3%) thought that oral contraceptives could increase the risk of developing breast cancer, 130 (16.3%) did not think that, and 266 (33.4%) were unsure (Table 2).

**Table 2:** Knowledge questions regarding risk factors of breast cancer by the study respondents (n = 796).

Variables	Yes	No	Not sure
Do you think breast cancer risk increases with advancing age?	**523 (65.7%**)	97 (12.2%)	176 (22.1%)
Do you think obesity increases the risk of developing breast cancer?	**329 (41.3%**)	268 (33.7%)	199 (25.0%)
Do you think irritation from a tight bra can increase breast cancer risk?	300 (37.7%)	**275 (34.5%**)	221 (27.8%)
Do you think having larger breasts increases the risk of developing breast cancer?	188 (23.6%)	**417 (52.4%**)	191 (24.0%)
Do you think alcohol consumption increases the risk of developing breast cancer?	**524 (65.8%**)	97 (12.2%)	175 (22.0%)
Do you think cigarette smoking increases the risk of developing breast cancer?	**581 (73.0%**)	90 (11.3%)	125 (15.7%)
Do you think early menarche (first menses) increases the risk of developing Breast cancer?	**218 (27.4%**)	353 (44.3%)	225 (28.3%)
Do you think late menopause (last menses) increases the risk of developing breast cancer?	**254 (31.9%**)	266 (33.4%)	276 (34.7%)
Do you think not breastfeeding increases the risk of developing breast cancer?	559 (70.2%)	**119 (14.9%**)	118 (14.8%)
Do you think positive family history increases the risk of developing breast cancer?	**641 (80.5%**)	60 (7.5%)	95 (11.9%)
Do you think oral contraceptives can increase the risk of developing breast cancer?	**400 (50.3%**)	130 (16.3%)	266 (33.4%)
Do you think hormone replacement therapy can increase the risk of developing breast cancer?	**446 (56.0%**)	71 (8.9%)	279 (35.1%)
Do you think radiation exposure can increase the risk of developing breast cancer?	**624 (78.4%**)	24 (3.0%)	148 (18.6%)

Bold categories represent correct answers.

### 3.3. Knowledge about symptoms of breast cancer

Five hundred forty seven (68.7%) of participants mentioned pain, 559 (70.2%) breast mass, 568 (71.3%) breast lumpiness, 583 (73.2%) nipple discharge, 416 (52.3) nipple ulcers, 437 (54.9%) color changes over the nipple, 325 (40.8%) breast warmth or itching, 427 (53.6%) redness or swelling of the breast, 352 (44.2%) skin thickening over breast, and 558 (70.1%) swollen axillary nodes as symptoms of breast cancer (Table 3).

**Table 3:** Symptoms that are suggestive of breast cancer according to study respondents (n = 796).

Symptoms reported	Descriptive/frequency
Pain	547 (68.7)
Breast mass	559 (70.2)
Breast lumpiness	568 (71.3)
Nipple discharge	583 (73.2)
Nipple ulcers	416 (52.3)
Color changes	437 (54.9)
Breast warmth or itching	325 (40.8)
Redness or swelling of the breast	427 (53.6)
Skin thickening over breast	352 (44.2)
Swollen axillary nodes	558 (70.1)

Data presented as frequency (%).

### 3.4. Attitude and perception towards mammography

Thirty two (4%) of the participants had undergone mammography at least once, and 764 (96%) had never undergone mammography. 26 (81.2%) were satisfied with mammography after having it, and 6 (18.8%) were unsatisfied. 222 (27.9%) had been counseled by a doctor about the advantages of undergoing mammography screening, and 574 (72.1%) were not counseled. 556 (69.8%) thought of undergoing mammography screening after seeing an advertisement on television, radio, or newspaper, 18 (2.3%) did not think to have it, and 222 (27.9%) were unsure.

Four hundred seventy one (59.2%) thought mammography is an effective tool to detect breast cancer, 449 (56.4%) thought that mammography was safe to perform, and 564 (70.9%) thought that mammography is a painful procedure. 30 (3.8%) thought that there is a cure for breast cancer, 481 (60.4%) did not think there is any cure for breast cancer, and 285 (35.8%) were unsure. 30 (3.8%) thought that mammography increases the risk of developing breast cancer, and 73 (21.7%) thought that mammography should be delayed until symptoms appear (Table 4).

**Table 4:** Attitude of the study participants towards screening mammography (n = 796).

Variables	Characteristics	Frequency (%)
Have you ever undergone mammography screening?	Yes	32 (4.0%)
	No	764 (96.0%)
If you have undergone mammography screening, can you share your experience with it? (n = 32)	Satisfied	26 (81.2%)
	Unsatisfied	6 (18.8%)
Have you ever been counseled by any doctor about the advantages of undergoing mammography screening?	**Yes**	**222 (27.9%**)
	No	574 (72.1%)
Have you ever thought of undergoing mammography screening after seeing an advertisement on TV, radio, or newspaper?	**Yes**	**556 (69.8%**)
	No	18 (2.3%)
	Unsure	222 (27.9%)
Do you think mammography is an effective tool for detecting breast cancer?	**Yes**	**471 (59.2%**)
	No	43 (5.4%)
	Unsure	282 (35.4%)
Do you think mammography is safe?	**Yes**	**449 (56.4%**)
	No	58 (7.3%)
	Unsure	289 (36.3%)
Do you think mammography is harmful?	Yes	158 (19.8%)
	**No**	**375 (47.1%**)
	Unsure	263 (33.0%)
Do you think mammography is a painful procedure?	**Yes**	**564 (70.9%**)
	No	66 (8.3%)
	Unsure	166 (20.9%)
Do you think there is a cure for breast cancer?	**Yes**	**30 (3.8%**)
	No	481 (60.4%)
	Unsure	285 (35.8%)
Do you think mammography increases the risk of developing breast cancer?	Yes	30 (3.8%)
	**No**	**481 (60.4%**)
	Unsure	285 (35.8%)
Should you delay mammography until you notice any symptoms?	Yes	173 (21.7%)
	**No**	**358 (45.0%**)
	Unsure	265 (33.3%)

Data are presented as frequency and percentages.

Bold categories represent correct answers.

According to regression analysis, higher education, higher monthly household income, and married marital status were significantly associated with good knowledge related to breast cancer (Table 5). Similarly, higher monthly household income was significantly associated with the positive attitudes of the study participants (Table 6).

**Table 5:** Regression analysis of factors associated with good knowledge among study respondents (score > 7) (n = 335).

Variables	Univariate	*P*-value	Multivariate	*P*-value
Age				
<20 yr	0.449 (0.216–0.930)	0.031*	0.927 (0.357–2.405)	0.876
20–30 yr	0.472 (0.251–0.886)	0.019*	0.801 (0.347–1.852)	0.604
30–40 yr	0.760 (0.373–1.547)	0.449	1.157 (0.529–2.531)	0.715
>40 yr	1.000	–	1.000	–
Education				
Primary up to matric (high school)	0.363 (0.144–0.917)	0.032*	0.290 (0.088–0.951)	0.041*
Up to secondary (intermediate)	0.843 (0.516–1.377)	0.495	0.570 (0.320–1.013)	0.055
Bachelors	0.647 (0.446–0.937)	0.021*	0.651 (0.427–0.992)	0.046*
Postgraduate	1.000	–	1.000	–
Residence				
Urban	1.000	–	–	–
Rural	1.079 (0.677–1.719)	0.751	–	–
Occupation				
Housewife	1.355 (0.865–2.123)	0.184	–	–
Student	1.011 (0.714–1.431)	0.952	–	–
Working women	1.000		–	–
Marital status				
Married	1.470 (1.060–2.037)	0.021*	0.572 (0.196–1.667)	0.306
Separated/widow	1.327 (0.620–2.841)	0.467	0.513 (0.140–1.880)	0.314
Single	1.000	–	1.000	–
Age of marriage				
<20 yr	1.000	–	–	–
20–30 yr	2.783 (1.293–5.986)	0.009*	2.168 (0.832–5.647)	0.113
>30 yr	2.068 (0.601–7.113)	0.249	1.518 (0.368–6.269)	0.564
Having children				
Yes	1.000	–	–	–
No	0.731 (0.512–1.043)	0.084	–	–
Age of menarche				
<12 yr	1.088 (0.720–1.644)	0.688	–	–
12–14 yr	1.329 (0.805–2.194)	0.266	–	–
>14 yr	1.000	–	–	–
Menopause				
Yes	1.170 (0.518–2.645)	0.706	–	–
No	1.000	–	–	–
Monthly household income				
<25,000 PKR	3.170 (1.990–5.051)	<0.001*	3.816 (2.213–6.579)	<0.001*
25,000–50,000 PKR	0.955 (0.615–1.483)	0.838	0.998 (0.631–1.577)	0.993
50,000–75,000 PKR	1.061 (0.692–1.625)	0.787	1.044 (0.673–1.620)	0.846
75,000–100,000 PKR	2.001 (1.333–3.004)	0.001*	2.140 (1.412–3.243)	<0.001*
>100,000 PKR	1.000	–	1.000	–
Any previous breast symptoms				
Yes	1.000	–	1.000	–
No	0.670 (0.458–0.981)	0.039*	0.648 (0.434–0.967)	0.034*
Any current breast symptoms				
Yes	1.000	–	–	–
No	1.099 (0.666–1.813)	0.711	–	–
Family history of breast cancer				
Yes	1.000	–	–	–
No	1.066 (0.752–1.511)	0.720	–	–

For knowledge questions (n = 13), each correct response carries 1 mark. The median knowledge score was 7.00. Hence good knowledge was considered as >7 scores out of 13.

PKR = Pakistani rupee.

* Significant *P*-values.

**Table 6:** Regression analysis of factors associated with a positive attitude among study respondents (score > 5) (n = 316).

Variables	Univariate	*P*-value	Multivariate	*P*-value
Age				
<20 yr	1.051 (0.502–2.202)	0.895	–	–
20–30 yr	1.064 (0.560–2.022)	0.849	–	–
30–40 yr	1.487 (0.722–3.062)	0.281	–	–
>40 yr	1.000	–	–	–
Education				
Primary to high school	0.769 (0.327–1.809)	0.548	–	–
Up to Secondary (Intermediate)	0.708 (0.429–1.169)	0.177	–	–
Undergraduate	0.788 (0.543–1.143)	0.209	–	–
Postgraduate	1.000	–	–	–
Residence				
Urban	1.000	–	–	–
Rural	1.138 (0.712–1.816)	0.589	–	–
Occupation				
Housewife	0.810 (0.513–1.276)	0.363	–	–
Student	0.854 (0.604–1.207)	0.371	–	–
Working women	1.000		–	–
Marital status				
Married	1.057 (0.759–1.472)	0.744	–	–
Separated/Widow	1.565 (0.732–3.345)	0.248	–	–
Single	1.000	–	–	–
Age of marriage				
<20 yr	1.000	–	–	–
20–30 yr	1.183 (0.570–2.455)	0.652	–	–
>30 yr	1.438 (0.428–4.833)	0.557	–	–
Having children				
Yes	1.000	–	–	–
No	0.902 (0.629–1.292)	0.572	–	–
Age of Menarche				
<12 yr	1.619 (0.972–2.697)	0.064	–	–
12–14 yr	1.245 (0.815–1.902)	0.311	–	–
>14 yr	1.000	–	–	–
Menopause				
Yes	1.088 (0.477–2.480)	0.841	–	–
No	1.000	–	–	–
Monthly household income				
<25,000 PKR	1.638 (1.034–2.595)	0.036*	2.020 (1.716–3.471)	0.011*
25,000–50,000 PKR	1.443 (0.935–2.225)	0.097	1.540 (0.978–2.426)	0.062
50,000–75,000 PKR	1.348 (0.880–2.066)	0.170	1.379 (0.887–2.142)	0.153
75,000–100,000 PKR	2.317 (1.538–3.491)	<0.001*	2.489 (1.632–3.796)	<0.001*
>100,000 PKR	1.000	–	1.000	–
Any previous breast symptoms				
Yes	1.000	–	–	–
No	0.904 (0.615–1.330)	0.609	–	–
Any current breast symptoms				
Yes	1.000	–	–	–
No	1.383 (0.822–2.327)	0.222	–	–
Family history of breast cancer				
Yes	1.000	–	–	–
No	0.709 (0.501–1.002)	0.052	–	–

For attitude questions (n = 11), each correct response carries 1 mark except for undergoing mammography and satisfying response (0.5 marks each). The median attitude score was 5.00. Hence positive attitude was considered as > 5 scores out of 10.

PKR = Pakistani rupee.

* Significant *P*-values.

## 4. Discussion

Breast cancer is a leading cause of cancer mortality in women causing about 15% of female cancer-related deaths. The incidence of breast cancer is shifting towards onset at a younger age. Knowledge and awareness are vital in early detection and optimal breast cancer treatment.^[9]^ This study aimed to evaluate Pakistani women’s knowledge about breast cancer awareness and screening procedures.

In this study, data was collected from women of all ages, with most of them belonging to the age group 20 to 30 years, with the majority having education level to intermediate living in urban areas. Most of the participants in the study were students, with most of the married women having children.^[15]^

The current study population had a suboptimal level of knowledge regarding breast cancer risk factors, with increasing age being a risk factor seen by 65.7% of the study population showing consistent results with a study conducted in 2021 by Pal et al^[16]^ in India as opposed to the results of a study by Santhanakrishnan et al^[17]^ on-nursing staff which reported it as 10.6%. 41.3% of the current study population believes that obesity is a risk factor for breast cancer which is higher than the results of a study conducted in 2015 in Lahore and is comparable to the results of a study by Al-Zalabani et al in Saudi Arabia.^[15,18]^ Most participants believed that wearing a tight brassiere increases the risk of developing breast cancer, but few also believed that having large breasts increases the risk. Chen et al^[19]^ reported that wearing a brassiere is not associated with breast cancer. Many study participants considered alcohol consumption (65.8%) and cigarette smoking (73%) as risk factors for breast cancer. These results were consistent with a study conducted in 2017 describing a significantly increased risk of breast cancer with smoking, particularly among women who started smoking at adolescent or premenarcheal ages.^[20]^ However, in a study conducted in Saudi Arabia, only 17.6% of women considered smoking a risk factor.^[15]^ Similarly, Warner et al^[21]^ have associated alcohol use with an increased risk of breast cancer, describing reducing alcohol consumption as a potential means to reduce breast cancer risk. Only 27.4% and 31.9% of the study population knew that early menarche and late menopause could increase the risk of breast cancer. Similar results were found in studies conducted in Jordan, Saudi Arabia, and India, showing a decreased awareness of these risk factors for breast cancer.^[15,22,23]^ Additionally, a majority (70.2%) of the current study population believes that not breastfeeding increases the risk of breast cancer. This is like the results of a study conducted in Jordan, which reported that 67.6% population had the same opinion.^[24]^ This is because of globalization and the adoption of Western lifestyles; many Pakistani women marry late, do not have first childbirth at an early age, and do not breastfeed for long, which increases the risk factor for breast cancer.^[18]^ Furthermore, the majority of the current study population has indicated oral contraceptives, hormone replacement therapy, and radiation exposure as risk factors for breast cancer which is consistent with the results of studies conducted in Saudi Arabia and Jordan.^[24,25]^ Consistent with many other studies, the current study found that the most known risk factor of breast cancer is a “family history of breast cancer” (80.5%).^[24–26]^ Therefore, breast cancer awareness campaigns should focus on other, less well-known, breast cancer risk factors, mainly since some of them can be influenced by lifestyle choices, such as exercise and obesity.

Our results regarding knowledge about signs and symptoms of breast cancer in the study population indicate that most participants are aware of the symptoms of breast cancer. Common symptoms are nipple discharge (73.2%), breast lump (71.3%), breast mass (70.2%), swollen lymph nodes (70. 1%), and pain (68.6%) being the most common. These results are similar to studies conducted in Saudi Arabia and Jordan.^[15,22]^ However, in studies conducted by Pal et al^[16]^ and Singh et al,^[27]^ nipple discharge and pain were reported by only 38.54%, 28.66%, 32.52%, and 25.45% of the study populations, respectively. Although awareness regarding breast lumps was seen in 62.29% of the study population by Pal et al.

Mammography is a gold standard technique for detecting breast cancer at its early stages. Regular mammography screening has been linked to decreased breast cancer mortality rates by 20% to 25%.^[28,29]^ The current study population was asked about their attitudes toward screening mammography. Out of which, only 4% of the study population had undergone mammography, with the majority being satisfied with the procedure. This is less than the study conducted by Al Mousa et al.^[22]^ The low proportion of women who had mammograms (5%) was similar to a Saudi study.^[30]^ Correspondingly, similar low proportions of mammography use were also reported in Nigeria, Malaysia, Iran, and Qatar.^[31–34]^ This may be because most of our study participants were younger belonging to the age group 20 to 30 years. And Pakistani women prefer not to visit a doctor until they feel worried about a specific sign or symptom. Therefore, extra efforts are needed to encourage them to take preventive measures toward breast cancer by increasing their knowledge about breast cancer risk factors, signs and symptoms, and early detection methods. Although only 4% of the study population had undergone mammography majority of the study population had a positive attitude (56.9%) toward this procedure. However, 60.4% felt that mammography increases the risk of breast cancer. Mammography in women with BRCA1 and BRCA2 mutations is not associated with an increased risk of breast cancer.^[35]^

This study’s strength lies in the comprehensive nature of the questionnaire encompassing a wide range of personal, cultural, and belief factors in addition to knowledge. Self-selection bias is a limiting factor in this study because most women were students and literate, which is not representative of the general population. Furthermore, there was no standardized questionnaire to measure breast cancer knowledge, so we created the questionnaire based on available information from previous studies. This limits the comparison of our results with other studies.

Breast cancer mortality is high in Pakistan because women present with advanced or end-stage disease.

## 5. Conclusions

Early breast cancer identification relies on awareness and knowledge. This study shows that despite knowing symptoms and risk factors, many neglect mammograms, likely due to misunderstanding their importance. A national screening program is essential, supported by stakeholders, to reduce stigma and promote screenings, especially among younger and rural women. Raising public awareness can encourage more screenings.

Our study assessed Pakistani women’s knowledge and attitudes toward breast cancer risks and screening. Participants, aged 20 to 30, identified various risk factors including increasing age, obesity, wearing tight bra, consuming alcohol and cigarettes, no breastfeeding, hormonal exposure, oral contraceptives, and radiation exposure but few underwent mammograms despite 60% recognizing their effectiveness. Increased awareness of breast cancer risks and the importance of mammography is needed among Pakistani women.

## Author contributions

**Conceptualization:** Qasim Mehmood, Abdul Rafay, Hafiza Qurat Ul Ain, Muhammad Junaid Tahir, Abdullah Saeed, Abdul Hanan, Zohaib Yousaf, Zair Hassan, Khabab Abbasher Hussien Mohamed Ahmed, Muhammad Sohaib Asghar.

**Data curation:** Qasim Mehmood, Hafiza Qurat Ul Ain, Muhammad Junaid Tahir, Abdullah Saeed, Abdul Hanan, Zohaib Yousaf, Muhammad Sohaib Asghar.

**Formal analysis:** Qasim Mehmood, Hafiza Qurat Ul Ain, Muhammad Junaid Tahir, Abdullah Saeed, Abdul Hanan, Zohaib Yousaf.

**Funding acquisition:** Qasim Mehmood, Hafiza Qurat Ul Ain, Abdullah Saeed.

**Investigation:** Qasim Mehmood, Hafiza Qurat Ul Ain.

**Methodology:** Qasim Mehmood, Abdul Rafay, Hafiza Qurat Ul Ain.

**Project administration:** Qasim Mehmood, Abdul Rafay, Hafiza Qurat Ul Ain.

**Resources:** Qasim Mehmood, Abdul Rafay.

**Software:** Qasim Mehmood, Abdul Rafay.

**Supervision:** Muhammad Junaid Tahir, Zohaib Yousaf, Muhammad Sohaib Asghar.

**Validation:** Qasim Mehmood, Abdul Rafay, Hafiza Qurat Ul Ain, Muhammad Junaid Tahir, Abdullah Saeed, Abdul Hanan, Zohaib Yousaf, Zair Hassan, Khabab Abbasher Hussien Mohamed Ahmed, Muhammad Sohaib Asghar.

**Visualization:** Qasim Mehmood, Abdul Rafay, Hafiza Qurat Ul Ain, Muhammad Junaid Tahir, Abdullah Saeed, Abdul Hanan, Zohaib Yousaf, Zair Hassan, Khabab Abbasher Hussien Mohamed Ahmed, Muhammad Sohaib Asghar.

**Writing – original draft:** Qasim Mehmood, Abdul Rafay, Hafiza Qurat Ul Ain, Muhammad Junaid Tahir, Abdullah Saeed, Abdul Hanan, Zohaib Yousaf, Zair Hassan, Khabab Abbasher Hussien Mohamed Ahmed, Muhammad Sohaib Asghar.

**Writing – review & editing:** Qasim Mehmood, Abdul Rafay, Hafiza Qurat Ul Ain, Muhammad Junaid Tahir, Abdullah Saeed, Abdul Hanan, Zohaib Yousaf, Zair Hassan, Khabab Abbasher Hussien Mohamed Ahmed, Muhammad Sohaib Asghar.
